# Detection of Core2 β-1,6-*N*-Acetylglucosaminyltransferase in Post-Digital Rectal Examination Urine Is a Reliable Indicator for Extracapsular Extension of Prostate Cancer

**DOI:** 10.1371/journal.pone.0138520

**Published:** 2015-09-21

**Authors:** Yuta Kojima, Tohru Yoneyama, Shingo Hatakeyama, Jotaro Mikami, Tendo Sato, Kazuyuki Mori, Yasuhiro Hashimoto, Takuya Koie, Chikara Ohyama, Minoru Fukuda, Yuki Tobisawa

**Affiliations:** 1 Department of Urology, Hirosaki University Graduate School of Medicine, Hirosaki, Japan; 2 Department of Advanced Transplant and Regenerative Medicine, Hirosaki University Graduate School of Medicine, Hirosaki, Japan; 3 Glycobiology Unit, Tumor Microenvironment Program, Cancer Center, Sanford-Burnham Medical Research Institute, La Jolla, CA 92037, United States of America; Queensland University of Technology, AUSTRALIA

## Abstract

To identify appropriate candidates for aggressive treatment such as radical prostatectomy or radiation therapy of localized prostate cancer (PCa), novel predictive biomarkers of PCa aggressiveness are essential. Core2 β-1,6-*N*-acetylglucosaminyltransferase-1 (GCNT1) is a key enzyme that forms core 2-branched *O*-glycans. Its expression is associated with the progression of several cancers. We established a mouse IgG monoclonal antibody (mAb) against GCNT1 and examined the relationship of GCNT1 expression to the clinicopathological status of PCa. Paraffin-embedded PCa specimens were analyzed by immunohistochemistry for GCNT1 expression using a newly established mouse anti-GCNT1 mAb by ourselves. GCNT1-positive tumor showed significantly higher Gleason score and larger tumor volume. The number of GCNT1-positive cases was significantly lower in cases of organ-confined disease than in cases of extracapsular extension. GCNT1-negative tumors were associated with significantly better prostate-specific antigen (PSA)-free survival compared with GCNT1-positive tumors. Multivariate analysis revealed that detection of GCNT1 expression was an independent risk factor for PSA recurrence. We established new methods for GCNT1 detection from PCa specimens. Immunoblotting was used to examine post-digital rectal examination (DRE) urine from PCa patients. Over 90% of GCNT1-positive PCa patients with high concentrations of PSA showed extracapsular extension. In conclusion, GCNT1 expression closely associates with the aggressive potential of PCa. Further research aims to develop GCNT1 detection in post-DRE urine as a marker for PCa aggressiveness.

## Introduction

In the United States and Europe, prostate cancer (PCa) is the most common malignancy in men and the second-leading cause of cancer-related death [1, 2]. The incidence of PCa is reportedly low in Asian countries [3]. However, its incidence is rapidly increasing in the Asia-Pacific region [4]. Some of the most critical issues related to PCa in clinical practice are overdiagnosis and overtreatment [5]. The lack of specificity of prostate-specific antigen (PSA) testing has resulted in a debate on the usefulness of PSA-based PCa screening [6, 7]. Moreover, unnecessary treatment for indolent PCa with a low malignant potential is a major issue, as aggressive PCa treatment is sometimes associated with adverse events. A promising alternate modality to prevent overtreatment is active surveillance [8]. However, the identification of suitable patients who are good candidates for aggressive treatment is associated with difficulties. To date, there are no perfect tools for precise detection of good candidate for active surveillance [9]. Therefore, the identification and validation of biomarkers of PCa aggressiveness are important in preventing PCa overtreatment.

Preoperative serum PSA levels and biopsy Gleason scores are conventional and powerful predictors of biological outcomes after radical prostatectomy for PCa [10, 11]. To improve the risk stratification for PCa recurrence after primary treatment in patients with localized PCa, many investigators have sought biomarkers that reflect the aggressive potential of PCa [12]. However, the majority of reported biomarkers have not been validated to provide information that is more useful than that provided by conventional clinicopathological parameters. With a novel biomarker representing the malignant potential of PCa, more accurate prediction of PSA recurrence and appropriate treatment selection may be possible.

Cell surface carbohydrate structures are altered during carcinogenesis and play important roles in cancer metastasis [13, 14]. The presence of sialyl Lewis X, which is one of the functional terminal structure, on the cell surface of colorectal cancer is positively correlated with poor prognosis [15]. In a similar way, high Gleason score prostate cancer specimens expressed sialyl Lewis X [16]. Branching glycan have increased a functional terminal structure, and a binding affinity for specific lectins [17]. In previous study, mannosyl (alpha-1,6-)-glycoprotein β -1,6-*N*-acetyl-glucosaminyltransferase (MGAT5) and Core 2 β -1, 6-*N*-acetylglucosaminyltransferase-1 (GCNT1) formed GlcNAc β1,6 branching glycan increased PCa aggressiveness [18, 19].

**Fig 1:**
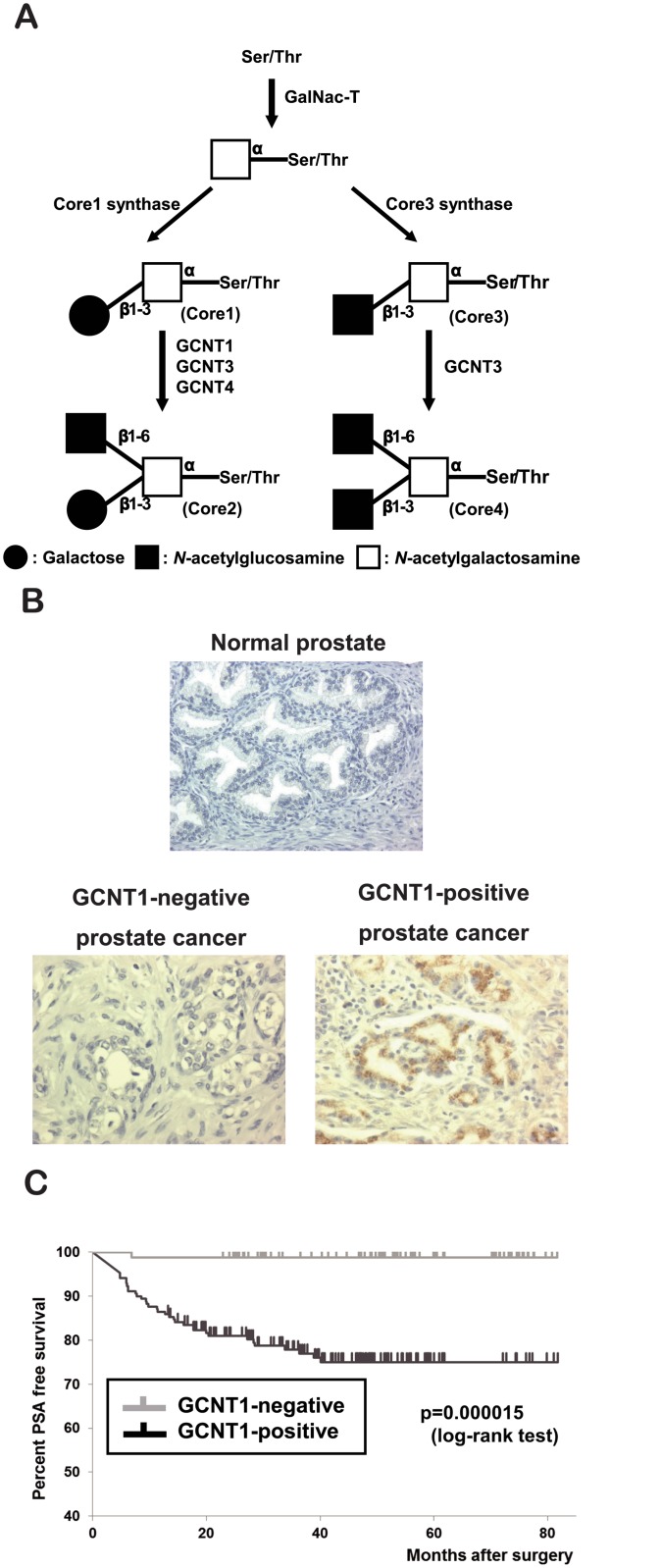
Core2 β-1,6-*N*-acetylglucosaminyltransferase-1 expression correlates with prostate cancer progression. (**A**) Biosynthetic pathways for *O*-glycans. (**B**) PCa specimens were incubated with an anti-core2 β-1,6-*N*-acetylglucosaminyltransferase-1 (GCNT1) monoclonal antibody (mAb), followed by a horseradish peroxidase (HRP)-conjugated secondary antibody. Counterstaining was performed using hematoxylin. GCNT1-positive cancer cells are brown. (**C**) Prostate-specific antigen-free survival periods were compared between GCNT1-positive and GCNT1-negative specimens. Survival was analyzed using Kaplan-Meier curves.

GCNT1 [20, 21] is a key enzyme that synthesizes core 2-branched *O*-glycans by catalyzing the transfer of *N*-acetylglucosamine from uridine diphosphate-*N*-acetylglucosamine with a β1, 6-linkage to α-*N*-acetylgalactosamine of a core 1 *O*-glycan (Fig 1A). A previous study analyzed *GCNT1* mRNA expression in fresh colorectal tumor samples and showed that expression of core 2-branched *O*-glycans is closely correlated with the malignant potential of colorectal cancer [22]. This is also true for pulmonary adenocarcinoma [23]. In immunohistochemistry using a polyclonal antibody [21], we demonstrated that GCNT1 expression is closely related with the aggressive potential of PCa [18], testicular cancer [24], and bladder cancer [25]. In recent study, a gene expression array showed GCNT1 was overexpressed in prostate cancer tissue [26]. However, the establishment of a monoclonal anti-GCNT1 antibody is essential for further research, including validation studies to elucidate the clinical utility of GCNT1 as a biomarker.

Here, we raised a monoclonal antibody (mAb) against GCNT1 by immunization of a mouse with GCNT1 specific peptide (S1 File) to evaluate the potential of the latter as an indicator of PCa aggressiveness. In this study, we demonstrated that the anti-GCNT1 mAb showed high specificity against human GCNT1 and that GCNT1 expression in PCa specimens from radical prostatectomy correlates with PCa aggressiveness. In addition, detection of GCNT1 in post-digital rectal examination (DRE) urine by the anti-GCNT1 mAb predicted extracapsular extension of PCa. Therefore, detection of GCNT1 in post-DRE urine may serve as a minimally invasive method to predict PCa aggressiveness.

## Materials and Methods

### Materials

ISOGEN II Reagent was purchased from Nippon Gene (Japan). A purified rabbit anti-mouse IgG antibody (γ-chain specific) was purchased from Zymed. A horseradish peroxidase (HRP)-conjugated goat anti-rabbit IgG (H+L) antibody and an HRP-conjugated goat anti-mouse IgG antibody were acquired from Cell Signaling Technology. An HRP-conjugated goat anti-mouse IgG antibody was acquired from Millipore. Purified mouse myeloma protein from MOPC 21 (IgG, κ), 2-mercaptoethanol, and bovine serum albumin (BSA) were purchased from Sigma-Aldrich. Tween-20 was purchased from Wako Pure Chemicals (Japan), as were DMEM and Ham’s F12 medium. Penicillin G/streptomycin solution was from Hyclone. Precision Plus Protein standards Dual Color were from Bio-Rad, and skim milk was from Yukijirushi (Japan).

### Cells

Chinese hamster ovary (CHO) cells were maintained in the alpha modification of Eagle's minimum essential medium (α-MEM) supplemented with 100 U/mL of penicillin, 100 μg/mL of streptomycin, and 10% fetal bovine serum (FBS).

### Immunohistochemical analysis of PCa specimens

Between 2005 and 2011, 250 PCa patients were treated with radical prostatectomy at the Department of Urology, Hirosaki University Graduate School of Medicine, Hirosaki, Japan. The tumor specimens were formalin-fixed and embedded in paraffin. Deparaffinized specimens were incubated with 5 μg/mL of mouse anti-human GCNT1 mAb (clone HU127), followed by incubation with HRP-conjugated goat anti-mouse IgG antibody (H+L; Millipore).

### Immunoblotting analysis of post-DRE urine specimens

Post-DRE urine was filled into 50 mL conical tubes, frozen immediately, and stored at -80°C until analysis. Post-DRE urine specimens were collected from 35 patients who underwent radical prostatectomy from 2010 to 2013 at the Department of Urology, Hirosaki University Graduate School of Medicine, Hirosaki, Japan. Frozen samples were thawed overnight at 4°C and briefly centrifuged (5000 x *g*, 5 min) to separate the supernatant and solids. Fifty microliters of the supernatant were spotted on to a nitrocellulose membrane. The membrane fixed with post-DRE urine protein was incubated with an anti-GCNT1 mAb (HU127), followed by an HRP-conjugated secondary antibody. Signals representing GCNT1 were enzymatically detected using the Novex® ECL Chemiluminescent Substrate Reagent Kit (Life Technologies) and visualized in a ChemiDocXRS+ System (Bio-Rad). Signal mean values were measured by Image Lab software (Bio-Rad). Amount of GCNT1 expression was calculated based on the signal mean values of recombinant human GCNT1 (R&D systems, 7248-GT). Auto-chemiliminescent signals were subtracted from total signals. Total protein concentration of post-DRE urine samples were measured by a BCA Protein Assay Kit (Pierce). Informed consent was obtained from all patients. All patients provided their written informed consent to participate in this study. The ethical committee of Hirosaki University approved the protocol of this study (The study about carbohydrate structure change in urological disease; Approval number: 2014–195). The study was performed in accordance with the ethical standards of the Declaration of Helsinki.

### Staging and grading of tumors

All patients were preoperatively evaluated using DRE, serum PSA testing, bone scanning, pelvic computed tomography, and transrectal ultrasonography. Using an 18-G needle, 6–12 prostate needle biopsy samples were obtained under ultrasound guidance. Staging was performed using the 2002 American Joint Committee on Cancer Staging Manual [27], while the Gleason grading system was used for tumor grading [28].

### PSA measurement and patient follow-up

Serum PSA levels were determined using IMx (Abbott Laboratories, Abbott Park, IL). Postoperative PSA levels were considered to be increased (PSA recurrence) if they were ≥ 0.2 ng/mL during two consecutive visits in a 1-month interval. Time zero was defined as the day of surgery. Patients with constantly detectable PSA levels (< 0.001 ng/mL) after surgery were recorded as recurrences at time zero. Follow-up intervals were calculated from the date of surgery to the last recorded follow-up (median, 48.4 months; range, 13.2–82.9 months).

### Statistical analysis

The chi-squared test was used to analyze the association of GCNT1 status with clinical and histopathological parameters. PSA-free survival was evaluated using Kaplan–Meier curves and differences between groups were assessed using the log-rank test. We used the SPSS 21.0 software package (SPSS, Chicago, IL) for all statistical analyses. Optimal PSA and GCNT1 expression cut-off values were calculated the using the following formula: cut-off = (1 − sensitivity)^2^ + (1 − specificity)^2^ [29, 30].

## Results

### GCNT1 expression in PCa positively correlates with cancer progression and PSA recurrence

To confirm antibody specificity for GCNT1, a mouse anti-human GCNT1 antibody was purified from hybridoma supernatant. Dose-dependent binding of the mouse anti-human GCNT1 mAb (clone HU127) to immobilized recombinant human GCNT1 (rhGCNT1) was detected using HRP-conjugated goat anti-mouse IgG in an enzyme-linked immunosorbent assay (ELISA) (S1A Fig). In the absence of immobilized rhGCNT1, no antibody binding was observed (S1B Fig). In immunoblotting analysis, the HU127 anti-human GCNT1 mAb bound specifically to rhGCNT1, but not to rhGCNT3 (S1C Fig). HU127 anti-human GCNT1 mAb also specifically detected transient expression of GCNT1 in CHO cells (S1D and S1E Fig). In the case of HU127 anti-human GCNT1 mAb premixed with GCNT1 peptide antigen prior to incubation on immunohistochemistry and immunoblotting, the GCNT1 signals were diminished (S1F and S1G Fig). The results also suggested that HU127 anti-human GCNT1 mAb held high specificity against GCNT1.

To evaluate the role of GCNT1 in PCa progression, PCa specimens were analyzed by immunohistochemistry using the HU127 anti-human GCNT1 mAb. The results demonstrated that GCNT1 was weakly expressed in healthy prostate glands. In contrast, some percentage of PCa cells expressed significant levels of GCNT1 (Fig 1B). When collated with these criteria, PCa specimens from 250 patients exhibited different clinical parameters (S1 Table). GCNT1 expression in prostatectomy specimens positively correlated with the postoperative Gleason score. Over 80% of tumor specimens with extracapsular extension of PCa (pT3 and pT4) expressed GCNT1, and GCNT1-positive tumors were significantly larger than GCNT1-negative tumors (S2 Table).

As shown in Fig 1C, GCNT1-positive patients were at significantly higher risk of PSA recurrence after radical prostatectomy. According to multivariate analysis, PSA levels, margin status, and GCNT1 expression in the tumor were independent risk factors for PSA recurrence (Table 1).

**Table 1:** Univariate and multivariable analyses of risk factors for prostate-specific antigen recurrence.

	Univariate	Multivariable	
	p-value	HR (95% CI)	p-value
**Age**	**0.053**	**1.004 (0.934–1.080)**	**0.905**
**iPSA** ^a^	**0.000**	**1.013 (0.990–1.037)**	**0.256**
**cT2b≤**	**0.182**	**0.637 (0.191–2.131)**	**0.464**
**bxGS** ^b^ **8≤**	**0.052**	**1.656 (0.612–4.482)**	**0.321**
**GS8≤**	**0.011**	**1.787 (0.720–4.435)**	**0.210**
**pT3≤** ^e^	**0.002**	**0.985 (0.349–2.774)**	**0.977**
**RM** ^c^	**0.000**	**4.966 (1.861–13.250)**	**0.001**
**pn** ^d^	**0.221**	**0.839 (0.315–2.234)**	**0.725**
**pN**	**0.000**	**29.124 (1.599–530.412)**	**0.023**
**GCNT1 status**	**0.028**	**3.691 (1.070–12.736)**	**0.039**

^a^, pre-treatment prostate-specific antigen

^b^, Gleason score

^c^, cancer existence at the resected margin

^d^, perineural invasion

^e^, extracapsular extension

CI, confidence interval; GCNT1, core2 β-1,6-*N*-acetylglucosaminyltransferase-1; HR, hazard ratio; PSA, prostate-specific antigen.

### Detection of GCNT1 in post-DRE urine of PCa patients allows prediction of extracapsular extension of PCa

**Fig 2:**
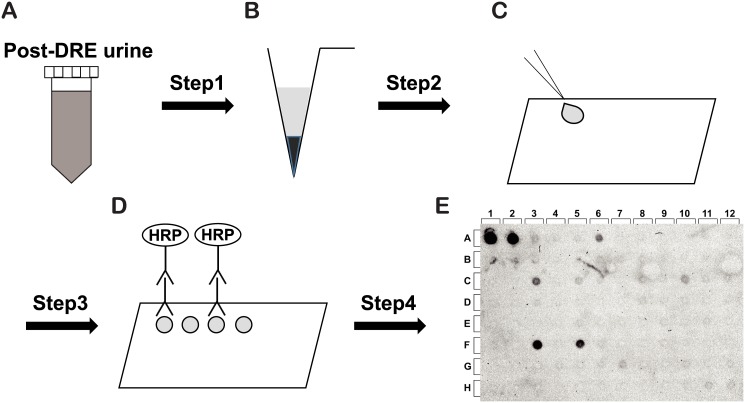
Detection of core2 β-1,6-*N*-acetylglucosaminyltransferase-1 in post-digital rectal examination urine specimens. (**A**) Post-digital rectal examination urine specimens were collected and (**B**) centrifuged. (**C**) Supernatants were collected and spotted onto a nitrocellulose membrane. (**D**) Core2 β-1,6-*N*-acetylglucosaminyltransferase-1 (GCNT1) was detected by an anti-GCNT1 monoclonal antibody, followed by a horseradish peroxidase (HRP)-conjugated antibody. (**E**) After treatment with a chemiluminescence reagent, the GCNT1 signal was recorded by a ChemiDoc+ system.

**Fig 3:**
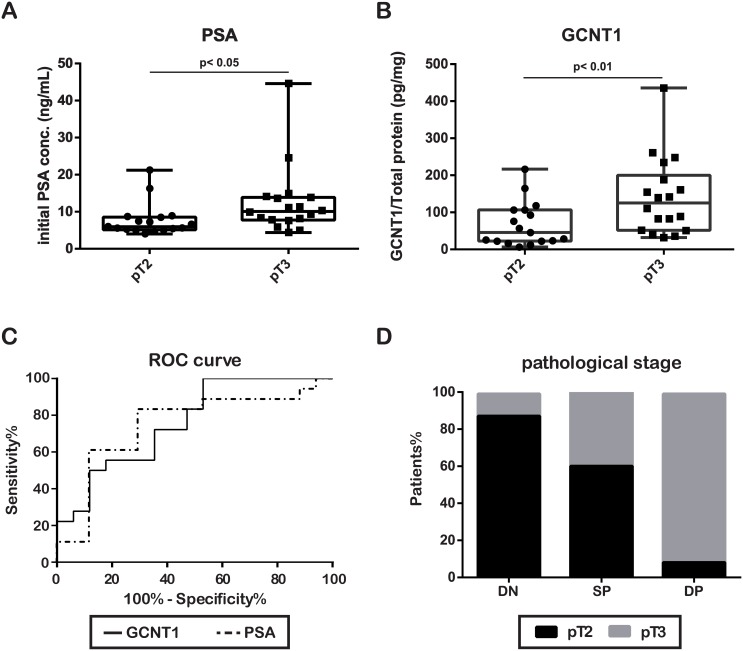
Prostate-specific antigen concentration and core2 β-1,6-*N*-acetylglucosaminyltransferase-1 expression predict extracapsular extension of prostate cancer. (**A**) Prostate-specific antigen (PSA) concentration and (**B**) Core2 β-1,6-*N*-acetylglucosaminyltransferase-1 (GCNT1) expression levels were significantly higher in prostate cancer (PCa) patients with extracapsular extension than in patients with organ-confined disease. (**C**) Receiver-operator characteristic curve analysis of PSA and GCNT1 revealed that the area under the curve of PSA was 0.7455 and GCNT1 was 0.7614. (**D**) Risk stratification was established using PSA and GCNT1 to predict the outcome of local PCa. Double negative (DN)-risk (PSA < 7.52 ng/mL, GCNT1< 79.36 pg/mg), single positive (SP)-risk (PSA > 7.52 ng/mL or GCNT1 > 79.36 pg/mg) and double positive (DP)-risk (PSA > 7.52 ng/mL and GCNT1 > 79.36 pg/mg) patients are compared.

To establish a semi-quantitative high-throughput screen for GCNT1 expression, post-DRE urine specimens, which contain high concentrations of PCa proteins, were analyzed by the dot-blotting method using the anti-human GCNT1 antibody (Fig 2). Prediction of extracapsular extension of PCa is a good predictor of PSA recurrence (S2 Fig). The initial PSA level and GCNT1 expression were highly correlated to extracapsular extension of PCa in a logistic regression analysis (Table 2). The optimal cut-off values for PSA and GCNT1 expression levels were determined to be 7.52 ng/mL and 79.36 pg/mg by the receiver-operator characteristic curve for prediction of extracapsular extension of PCa using the following formula: cut-off = (1 − sensitivity)^2^ + (1 − specificity)^2^ [29, 30] (Fig 3A–3C). Based on these clinicopathological parameters, we established following recurrence risk stratifications: double negative-risk (PSA < 7.52 ng/mL, GCNT1 < 79.36 pg/mg), single positive-risk (PSA > 7.52 ng/mL or GCNT1 > 79.36 pg/mg) and double positive-risk (PSA > 7.52 ng/mL and GCNT1 > 79.36 pg/mg). Over 90% of double positive-risk patients had extracapsular extension of PCa in this risk stratification (Fig 3D). These results indicate that a high PSA concentration and GCNT1 expression in post-DRE urine are good predictors of extracapsular extension of PCa.

**Table 2:** Logistic regression analyses of risk factors for extracapsular extension of prostate cancer.

	HR (95% CI)	p-value
**Age**	**1.003 (0.823–1.222)**	**0.978**
**iPSA** ^a^	**1.252 (0.984–1.594)**	**0.068**
**cT2b≤**	**0.718 (0.015–33.566)**	**0.866**
**bxGS** ^b^ **8≤**	**1.569 (0.457–5.385)**	**0.474**
**GCNT1 status**	**1.031 (1.009–1.053)**	**0.006**

^a^; pre-treatment prostate-specific antigen

^b^; Gleason score

CI, confidence interval; GCNT1, core2 β-1,6-*N*-acetylglucosaminyltransferase-1; HR, hazard ratio; PSA, prostate-specific antigen

## Discussion

Aberrant glycosylation of cell surface glycoproteins plays an important role in cancer initiation, proliferation, invasion, and metastasis [31–33]. Biosynthesis of oligosaccharides on glycoproteins is performed in concert by several glycosyltransferases. The functional terminal structure such as sialy lewis X (sLeX) and sialyl lewis A (sLeA) is also formed by glycosyltransferases [16, 34]. The sLeX and sLeA, which were widely known ligand of carbohydrate-binding proteins, are closely related to metastasis [35]. Not only sLe antigens, but also internal structures, particularly GlcNAc beta1,6 branching structures and polylactosamines, are closely related to cancer malignancy [22, 31]. GCNT1 is one of the glycosyltransferases that forms the core 2 *O*-glycans on the surface of lymphocytes and various cancer cells [18, 24, 36, 37]. Previously, it was reported that GCNT1 expression is associated with the metastatic potential of colorectal cancer [22], lung cancer [23], testicular cancer [24] and PCa [18]. It has also been reported that GCNT1-expressing cancers can escape the host immune response [25, 38], especially from host natural killer cells that bind to galectin-3 on core 2-branching *O*-glycans [25, 38].

This study established a mAb against GCNT1 and evaluated its potential as an indicator of PCa aggressiveness. Immunohistochemical analysis of radical prostatectomy specimens showed that GCNT1 expression on PCa cells closely related to extracapsular extension of PCa (Fig 1). Moreover, patients with GCNT1-positive PCa exhibited worse PSA-free survival compared with patients with GCNT1-negative tumors (Fig 1). These results indicate that GCNT1 expression strongly correlates with the malignant potential of PCa.

Although immunohistochemistry provided much information on protein expression in PCa, the analysis was not quantitative. Using post-DRE urine, we established a semi-quantitative and high-throughput screening for the malignant potential of PCa (Figs 2 and 3). Recent studies reported that prostate cancer antigen 3 and a TMPRSS2:ERG fusion were two of the most useful indicators of PCa. These markers were focused on prospective PCa screening and early detection of PCa [39]. Prostate cancer antigen 3 and the TMPRSS2:ERG fusion almost reported PCR-based study and did not present sufficient biological evidence of PCa aggressiveness. We also reported that detection of aberrant glycosylation of PSA improved PCa screening but did not predict PCa aggressiveness [40]. In this study, GCNT1 expression in post-DRE urine was correlated with extracapsular extension of PCa. Moreover, a combination of initial PSA concentration and GCNT1 expression could predict extracapsular extension in over 90% of PCa. Therefore, GCNT1 detection in post-DRE urine improved prediction of PCa invasiveness.

Because GCNT1 is not a cancer-specific protein, its expression was unsuitable for PCa screening. Therefore, a combination of reported PCa screening markers and GCNT1 may improve the development of therapeutic strategies of PCa. Although the mechanism of GCNT1-driven regulation in cancer progression is poorly understood, our study demonstrates that GCNT1 can be a predictor of the malignant potential of PCa. Further clinical research is necessary to determine the utility of GCNT1 as a biomarker of PCa.

## Supporting Information

S1 Fig Preparation of an anti-human core 2 β-1,6-*N*-acetylglucosaminyltransferase-1 monoclonal antibody. (**A**) Binding of the core2 β-1,6-*N*-acetylglucosaminyltransferase-1 (GCNT1)-specific antibodies. Culture supernatants were prepared from HU127 hybridoma cells. Binding of the anti-human GCNT1 monoclonal antibody (mAb, blue line) or IgG myeloma MOPC 21 (control, orange line) to immobilized recombinant human GCNT1 in a concentration-dependent manner (abscissa) was detected using a horseradish peroxidase (HRP)-conjugated anti-mouse IgG antibody. Error bars indicate the standard deviation of triplicate measurements. Concentrations of the antibody in supernatants were determined using a sandwich ELISA. The results are representative of two experiments. (**B**) The anti-human GCNT1 mAb recognized immobilized recombinant human GCNT1, but not BSA. (**C**) To confirm anti-human GCNT1 mAb specificity, recombinant human GCNT1 and GCNT3 were analyzed using electrophoresis (SDS-PAGE) and transferred to a PVDF membrane. The anti-human GCNT1 mAb specifically recognized GCNT1, but not GCNT3. (**D**) Immunoblotting and (**E**) immunocytochemistry revealed the anti-human GCNT1 mAb also specifically recognized GCNT1 in GCNT1-overexpressed CHO cells. Peptide inhibition assay for (F) IHC and (G) dot-blotting methods revealed the GCNT1 signals were inhibited by GCNT1 antigen peptide pre-treated anti-human GCNT1 mAb.
(TIF)

S2 Fig Extracapsular extension of prostate cancer was one of the strong predictor of prostate-specific antigen recurrence. Prostate-specific antigen-free survival periods were compared between organ-confined disease (pT2) and extracapsular extension (pT3 and pT4). Survival was analyzed by Kaplan-Meier curves.
(EPS)

S1 File Supplementary materials and methods. (DOCX)

S1 Table Core2 β-1,6-*N*-acetylglucosaminyltransferase-1 status and patient data. (DOCX)

S2 Table Core2 β-1,6-*N*-acetylglucosaminyltransferase-1 status and pathological parameters. (DOCX)

S3 Table Patient data of post-digital rectal examination urine specimens. (DOCX)

## Abbreviations

DRE: digital rectal examination
GCNT1: core2 β-1,6-*N*-acetylglucosaminyltransferase-1
HRP: horseradish peroxidase
mAb: monoclonal antibody
PCa: prostate cancer
PSA: prostate-specific antigen
RLU: relative light units
