# Supplementary figures and images for: Detection of Core2 β-1,6-N-Acetylglucosaminyltransferase in Post-Digital Rectal Examination Urine Is a Reliable Indicator for Extracapsular Extension of Prostate Cancer
Source: PLoS One. 2015 Sep 21;10(9):e0138520. doi: 10.1371/journal.pone.0138520 (PMC4577128; doi:10.1371/journal.pone.0138520)

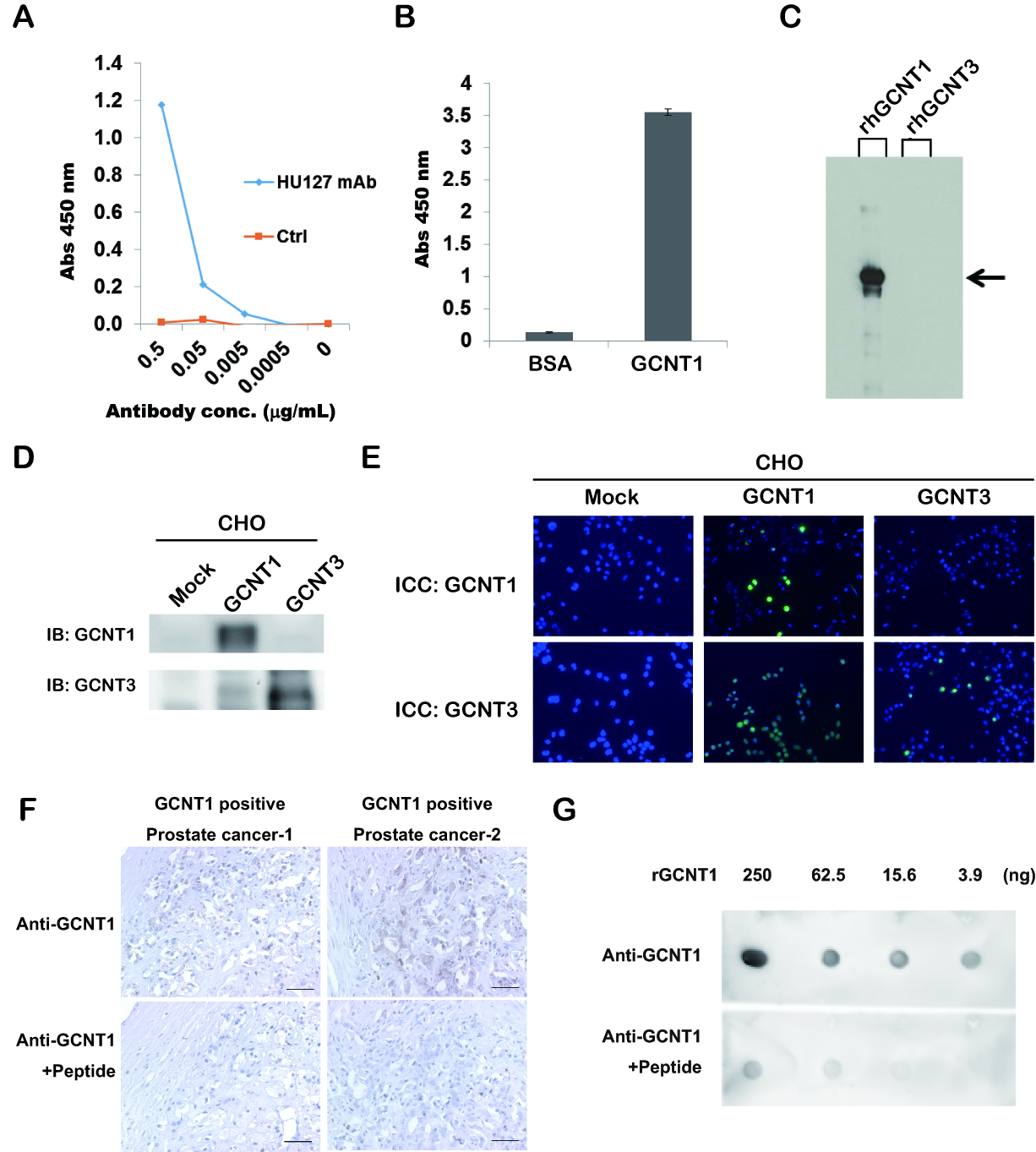

Supplement: S1 Fig — (A) Binding of the core2 β-1,6-N-acetylglucosaminyltransferase-1 (GCNT1)-specific antibodies. Culture supernatants were prepared from HU127 hybridoma cells. Binding of the anti-human GCNT1 monoclonal antibody (mAb, blue line) or IgG myeloma MOPC 21 (control, orange line) to immobilized recombinant human GCNT1 in a concentration-dependent manner (abscissa) was detected using a horseradish peroxidase (HRP)-conjugated anti-mouse IgG antibody. Error bars indicate the standard deviation of triplicate measurements. Concentrations of the antibody in supernatants were determined using a sandwich ELISA. The results are representative of two experiments. (B) The anti-human GCNT1 mAb recognized immobilized recombinant human GCNT1, but not BSA. (C) To confirm anti-human GCNT1 mAb specificity, recombinant human GCNT1 and GCNT3 were analyzed using electrophoresis (SDS-PAGE) and transferred to a PVDF membrane. The anti-human GCNT1 mAb specifically recognized GCNT1, but not GCNT3. (D) Immunoblotting and (E) immunocytochemistry revealed the anti-human GCNT1 mAb also specifically recognized GCNT1 in GCNT1-overexpressed CHO cells. Peptide inhibition assay for (F) IHC and (G) dot-blotting methods revealed the GCNT1 signals were inhibited by GCNT1 antigen peptide pre-treated anti-human GCNT1 mAb. (TIF) [file pone.0138520.s001.tif]
