# Supplementary material for: Detection of Core2 β-1,6-N-Acetylglucosaminyltransferase in Post-Digital Rectal Examination Urine Is a Reliable Indicator for Extracapsular Extension of Prostate Cancer
Source: PLoS One. 2015 Sep 21;10(9):e0138520. doi: 10.1371/journal.pone.0138520 (PMC4577128; doi:10.1371/journal.pone.0138520)
Supplement: S1 File — (DOCX) [file pone.0138520.s003.docx]

**S1 File. Supplementary materials and methods**

*Cells.* Nonsecreting mouse myeloma P3U1 cells were purchased from the American Type Culture Collection (Rockville, MD, USA).

*Immunization and production of hybridoma cells.* To generate antibodies, 2 mg/mL of a keyhole limpet hemocyanin (KLH)-conjugated peptide antigen (GCNT1 N240-V260) was mixed with an equal amount of Titer Max Gold adjuvant (Sigma–Aldrich). BALB/C mice were immunized twice intraperitoneally with a two-week interval between immunizations. Each mouse received 100 µg of the KLH-conjugated peptide antigen and Titer Max Gold mixture. The immune responses of the mice were boosted with an intraperitoneal injection of 40 µg of the KLH-conjugated antigen peptide.

The mice were sacrificed by cervical dislocation three days after the booster immunization. Cell suspensions were prepared from the popliteal lymph node, iliac lymph node, and spleen. Lymphocytes were fused with P3U1 myeloma cells using PEG1500 (Roche, Indianapolis, IN) at 37°C. Fused cells were cultured in HAT medium containing 20% FBS and incubated at 37°C in a humidified atmosphere containing 5% CO_2_. The culture medium was gradually replaced with HT medium containing 20% FBS and ALyS-Basal medium supplemented with 5% HLCM (Cell Science & Technology Institute, Sendai, Japan). Hybridoma cells secreting specific antibodies were cloned and recloned using the limiting dilution method. To obtain serum-free mAb samples, cloned hybridoma cells were cultured in ALyS-Ab Pro medium.

To screen hybridomas and assess antibody binding to GCNT1, GCNT1-specific IgG antibodies in each sample were quantified by ELISA. Briefly, binding of IgG to an immobilized ovalbumin-conjugated antigen (1 μg/mL in PBS) on a 96-well microtiter plate was determined by incubation with a 1:2500 dilution of an alkaline phosphatase-conjugated goat anti-mouse IgG antibody (γ-chain specific). After an alkaline phosphatase substrate was added, absorbance at 495 nm in each well was measured using a microplate reader.

*Construction of the expression vector.* Coding sequences of GCNT1- and GCNT3-specific primers were as follows. hC2GnT1-SC-Nde-F1; 5’- TTTCATATGAGGATTCATCAAAAGCCT-3’, hC2GnT1-Xho-R1; 5’- TTTCTCGAGTCAGTGTTTTAATGTCTCCA-3’, hC2GnT2-SC-Nde-F1; 5’- TTTCATATGCTGAAACTTTCTTTCAGGTTGAAG-3’, and hC2GnT2-SC-Xho-R1; 5’- TTTCTCGAGTCAAAGTTCAGTCCCATAGATGG-3’ were used to amplify GCNT1 and GCNT3 cDNA, respectively, by PCR to construct a bacterial expression construct using the pCold-TF DNA vector (TaKaRa, Japan). PCR products were purified from agarose gels, sequentially digested with *Nde*I and *Xho*I, and ligated into the pCold-TF DNA vector.

To construct a mammalian expression vector, the coding sequence of GCNT1 was amplified by PCR using the primer pair hC2GnT-BamH-Full-F1; 5’- TTTGGATCCTGCCCTTCACAAAGGAAATC-3’ and hC2GnT-Not-R1; 5’- TTTGCGGCCGCGCCCGTAATGGTCAGTGTTT-3’. The resulting PCR product was digested with *Bam*HI and *Not*I restriction enzymes and ligated into pEBmulti-Neo (Wako Pure Chemicals).

*Determination of monoclonal antibody specificity to GCNT1 using rhGCNT1.* rhGCNT1 or BSA were adsorbed to the well walls of a 96-well microtiter plate. The wells were then blocked with a BSA solution in PBS and hGCNT1 mAbs were added to each well. Specific binding to GCNT1 was determined by incubation with a 1:2000 dilution of a HRP-conjugated goat anti-mouse IgG antibody (H+L). After an HRP substrate was added to each well, absorbance at 405 nm was measured on a microplate reader.

For immunoblotting, 1 mg/mL rhGCNT1 or GCNT3 was applied on an SDS-PAGE gel. After electrophoresis, proteins were transferred to a PVDF membrane. The membrane was blocked using 5% skim milk in TBS. GCNT1 was detected by sequential incubation with an anti-rhGCNT1 mAb (clone HU127, 5 μg/mL) and an HRP-goat anti-mouse IgG antibody (H+L; 1:2000) diluted in 0.05% Tween-20 in TBS (TBST). Signals representing GCNT1 were enzymatically detected using the ECL Plus reagent (GE Healthcare).

*Determination of the binding specificity of anti-GCNT1 mAb by immunoblotting and immunocytochemistry*

CHO cells were maintained in α-MEM supplemented with 10% FBS. Cells were plated in a 6-well cell culture dish (Thermo Fisher) 24 h before transfection. Cells were transfected using the XtremeGENE HP DNA transfection reagent (Roche Diagnostics) with 1 µg of GCN1T, GCNT3, or an empty vector (mock). For immunoblotting, the cells were lysed by 1% NP-40 in PBS 72 h post-transfection and 3 µg proteins from the CHO whole-cell lysate were applied on an SDS-PAGE gel. After electrophoresis, proteins were transferred onto a PVDF membrane. Unspecific binding was blocked with 5% BSA in TBST. Next, GCNT1 and GCNT3 were detected using sequential incubation with anti-GCNT1 mAb (clone HU127) or anti-GCNT3 antibody (Sigma) and HRP-conjugated secondary antibodies (1:2000) diluted in 5% BSA in TBST. Signals representing GCNT1 and GCNT3 were enzymatically detected using the Novex^®^ ECL Chemiluminescent Substrate Reagent Kit (Life Technologies). For immunocytochemistry, CHO cells were cultured on glass coverslips and transfected by using the XtremeGENE HP DNA transfection reagent (Roche). Cells were fixed by 4% paraformaldehyde in PBS. Coverslips were incubated with primary antibodies and Alexa488-conjugated secondary antibodies. Nuclei was stained by DAPI and cells were visualized by fluorescence microscopy (EZ-9000, Keyence, Osaka, Japan).

To confirm the binding specificity of anti-GCNT1 mAb under immunohistochemistry and dot blotting analysis, anti-GCNT1 mAb was premixed with GCNT1 peptide antigen (GCNT1 N240-V260). For immunohistochemistry, GCNT1 positive specimens were incubated with 5 μg/mL of mouse anti-human GCNT1 mAb (clone HU127) which were premixed with BSA or 50 μg/mL GCNT1 peptide antigen, followed by incubation with HRP-conjugated goat anti-mouse IgG antibody (H+L; Millipore). After washing, colored reaction products were developed using an EnVision+ System-HRP (DAB; Dako) and sections were counterstained with hematoxylin solution. For dot-blotting methods, rGCNT1 (250, 62.5, 15.6 and 3.9 ng) spotted on to a nitrocellulose membrane. The membrane fixed with rGCNT1 were incubated with 1 μg/mL of mouse anti-human GCNT1 mAb (clone HU127) which were premixed with BSA or 10 μg/mL GCNT1 peptide antigen, followed by an HRP-conjugated secondary antibody. Signals representing GCNT1 were enzymatically detected using the Novex® ECL Chemiluminescent Substrate Reagent Kit (Life Technologies) and visualized in a ChemiDocXRS+ System (Bio-Rad).
