# Supplementary material for: Detection of Core2 β-1,6-N-Acetylglucosaminyltransferase in Post-Digital Rectal Examination Urine Is a Reliable Indicator for Extracapsular Extension of Prostate Cancer
Source: PLoS One. 2015 Sep 21;10(9):e0138520. doi: 10.1371/journal.pone.0138520 (PMC4577128; doi:10.1371/journal.pone.0138520)
Supplement: S1 Table — (DOCX) [file pone.0138520.s004.docx]

**S1 Table.** **Core2 β-1,6-*N*-acetylglucosaminyltransferase-1 status and patient data.**

|  | GCNT1-negative | GCNT1-positive | p-value | |
| --- | --- | --- | --- | --- |
| Number of Patients | **80** | **170** | |  |
| Age (years old)^a^ | **67.76 ± 5.18** | **66.82 ± 5.38** | | **0.096** |
| PSA^a, b^ | **8.23 ± 4.30** | **9.48 ± 6.17** | | **0.051** |
| bx^c^ Gleason score (GS)^a^ | **67.76 ± 5.18** | **67.76 ± 5.18** | | **0.134** |
| Clinical stage (cT) (%)^d^ |  |  | | **0.859** |
| cT1 | **52/159 (32.7%)** | **107/159 (67.3%)** | |  |
| cT2 | **25/79 (31.6%)** | **54/79 (68.4%)** | |  |
| cT3 | **3/11 (27.3%)** | **8/11 (72.7%)** | |  |
| cT4 | **0/1 (0%)** | **1/1 (100%)** | |  |

a, Statistical analysis by Student’s t-test; b, pre-treatment prostate-specific antigen; c, biopsy; d, statistical analysis by chi-squared test; GCNT1, core2 β-1,6-*N*-acetylglucosaminyltransferase-1; PSA, prostate-specific antigen.
