# Supplementary material for: Detection of Core2 β-1,6-N-Acetylglucosaminyltransferase in Post-Digital Rectal Examination Urine Is a Reliable Indicator for Extracapsular Extension of Prostate Cancer
Source: PLoS One. 2015 Sep 21;10(9):e0138520. doi: 10.1371/journal.pone.0138520 (PMC4577128; doi:10.1371/journal.pone.0138520)
Supplement: S2 Table — (DOCX) [file pone.0138520.s005.docx]

**S2 Table. Core2 β-1,6-*N*-acetylglucosaminyltransferase-1 status and pathological parameters.**

|  | GCNT1-negative | GCNT1-positive | p-value | |
| --- | --- | --- | --- | --- |
| Number of Patients | **80** | **170** | |  |
| GS^a, b^ | **7.30 ± 0.74** | **7.54 ± 0.88** | | **0.017** |
| Final pathological stage (pT) (%)^c^ | |  | | **0.003** |
| pT2 | **61/153 (39.9%)** | **92/153 (60.1%)** | |  |
| pT3 | **19/96 (19.8%)** | **77/96 (80.2%)** | |  |
| pT4 | **0/1 (0%)** | **1/1 (100%)** | |  |
| Tumor volume (cm^3^)^b^ | **0/1 (0%)** | **1/1 (100%)** | | **0.000** |

a, Gleason score; b, statistical analysis by Student’s t-test; c, statistical analysis by chi-squared test; GCNT1, core2 β-1,6-*N*-acetylglucosaminyltransferase-1
