# Supplementary material for: Detection of Core2 β-1,6-N-Acetylglucosaminyltransferase in Post-Digital Rectal Examination Urine Is a Reliable Indicator for Extracapsular Extension of Prostate Cancer
Source: PLoS One. 2015 Sep 21;10(9):e0138520. doi: 10.1371/journal.pone.0138520 (PMC4577128; doi:10.1371/journal.pone.0138520)
Supplement: S3 Table — (DOCX) [file pone.0138520.s006.docx]

**S3 Table. Patient data of post-digital rectal examination urine specimens.**

| Number of Patients |  | 35 |  |
| --- | --- | --- | --- |
| Age (years old)^a^ |  | **67.57 ± 5.32** |  |
| PSA^a, b^ |  | **10.17 ± 7.63** |  |
| bx^c^ Gleason score (GS)^a^ | |  |  |
| 7 | | **21** | **(60.0%)** |
| 8≤ | | **14** | **(40.0%)** |
| Clinical stage (cT) (%) | |  |  |
| cT1c | | **23** | **(65.7%)** |
| cT2a |  | **5** | **(14.3%)** |
| cT2b |  | **3** | **(8.6%)** |
| cT2c |  | **2** | **(5.7%)** |
| cT3 |  | **2** | **(5.7%)** |

a, statistical analysis by Student’s t-test; b, pre-treatment prostate-specific antigen; c, biopsy; PSA, prostate-specific antigen
